# Liquid Biopsies in Head and Neck Cancer: Current State and Future Challenges

**DOI:** 10.3390/cancers13081874

**Published:** 2021-04-14

**Authors:** Lingyi Kong, Andrew C. Birkeland

**Affiliations:** 1 The First Clinical College of Tongji Medical College, Huazhong University of Science and Technology, Wuhan 430030, China; u201712215@hust.edu.cn; 2 Department of Otolaryngology-Head and Neck Surgery, University of California, Davis, CA 95817, USA

**Keywords:** head and neck cancer, liquid biopsy, circulating tumor cells, circulating tumor DNA, exosomes, head and neck squamous cell carcinoma

## Abstract

**Simple Summary:**

This review covers the most recent and up-to-date literature on liquid biopsy techniques in head and neck cancer. Head and neck cancer remains a common, difficult to treat and deadly type of disease. Part of the challenge in treating head and neck cancer lies in identifying disease at its early stages, determining responses to treatment and managing any recurrent disease. Liquid biopsies are the sampling of body fluids to determine markers that can indicate the presence of cancer. These technologies have immense potential in helping improve treatment for head and neck cancers. In this review, we discuss the current state of liquid biopsies for head and neck cancer, as well as limitations and challenges the field must overcome to advance our research in this area.

**Abstract:**

Head and neck cancers are the seventh most frequent malignancy worldwide, consisting of a heterogeneous group of cancers that develop in the oral cavity, pharynx, and larynx, with head and neck squamous cell carcinoma (HNSCC) being the most common pathology. Due to limitations with screening and physical examination, HNSCC often presents in advanced disease states and is thus associated with poor survival. In this setting, liquid biopsies, or obtaining patient bodily fluid samples for cancer diagnosis and prognosis, may play a dramatic role in optimizing care for HNSCC patients. In recent years, there have been dramatic advancements in investigations focused on optimizing and implementing liquid biopsies in general, and specifically for HNSCC patients. Moving forward, there remain significant challenges in liquid biopsy technological development, as well as opportunities for the development of HNSCC liquid biopsy clinical trials and treatment paradigms. In this review, we discuss the current state of liquid biopsy technologies via circulating tumor cells, circulating tumor DNA and exosomes, approaches in head and neck cancer, challenges to optimization and application of liquid biopsies for clinical study, and future prospects for this field of research as it applies to head and neck cancer.

## 1. Introduction

Head and neck cancer is the seventh most common cancer worldwide, with 890,000 new cases and 450,000 deaths in 2018 [1], representing 3% of all cancers and slightly above 1.5% of all cancer deaths in the United States [2]. Head and neck squamous cell carcinomas (HNSCC) are by far the most common form of head and neck cancer, arising from mucosal surfaces of the oral cavity, pharynx, and larynx. Head and neck squamous cell carcinomas have been historically found in older patients with heavy tobacco and alcohol use; recently, there has been a declining global trend in traditional HNSCC cases due to decreased use of tobacco [3,4], counterbalanced with increased cases of HPV-associated oropharyngeal cancer (primarily by HPV type 16) [4,5].

In the era of exome- and genome-wide sequencing, tumor molecular profiling has been important in better understanding the diagnosis, treatment and prognosis of HNSCC in clinical practice. Currently, analysis based on tissue biopsy or cytology samples is still the gold standard in diagnosis of HNSCC. However, tissue biopsy may involve invasive procedures, often in a general anesthetic surgical setting. Thus, there are limitations in tissue access and repeatability of tumor sampling with current biopsy techniques. As there are considerable benefits to repeated biopsies both spatially (to account for tumor heterogeneity) and temporally (to account for dynamic tumor evolution), new methodologies for cancer diagnosis and assessment are needed. Over the last decade, there has been increasing interest in using liquid biopsies to detect cancer-specific biomarkers in patients’ body fluids [6,7]. Liquid biopsy has been reported to play roles in early malignancy detection in diverse tumor types [8,9]. As a rapid and noninvasive approach, liquid biopsies have emerged as an exciting investigational avenue to obtain information on cancer diagnosis, treatment response, and progression [10,11].

In general, liquid biopsy refers to analytes from various biological fluids, such as blood or other accessible fluids: ascites, pleural effusions, saliva, or urine. Most liquid biopsy-based diagnostic tests for solid malignancies are based on blood serum or plasma specimens [6,7]. In studies of HNSCC, saliva is an additional medium for detection of tumor biomarkers [12]. Currently, the most common analytes used for liquid biopsy include circulating tumor cells (CTCs), cell-free tumor DNA (ctDNA), proteins, metabolites, exosomes (EXOs), mRNA, and miRNAs. In this review, we will focus on the applications of CTCs, ctDNA, and EXOs in head and neck cancer diagnosis, response to therapy, and prognosis. Additionally, we will: (i) review the current state of literature using these biomarkers in head and neck cancer, (ii) summarize current technological challenges and (iii) provide an outlook on future prospects in this rapidly evolving field.

## 2. Circulating Tumor Cells in Head and Neck Cancer

### 2.1. Characterization and Detection Techniques

Circulating tumor cells (CTCs) represent transient cancer cells originating from a primary tumor or metastatic sites that have the capacity to enter the adjacent vasculature and disseminate to distant sites [13]. Notably, CTCs are extremely rare, with approximately 1–100 CTC/mL among billions of red blood cells [14], making it challenging to detect and capture CTCs from whole blood samples. 

Different methods have been established to isolate CTCs, which can be broadly classified as either label-dependent (a positive selection of CTCs making use of specific markers expressed by tumor cells, such as epithelial cell adhesion molecules (EpCAM), cytokeratins (CKs) and integrins) [15] or label-independent (based on physical property differences such as size and density between CTCs and surrounding blood cells) [16]. Integrins, being cell surface receptors highly expressed in many different tumor types, have been an intriguing target for isolating CTCs. Some researchers have applied tumor cell specific ligands, such as LXY30 [15], targeting alpha 3 beta1 integrin, to enrich lung cancer CTCs from patient blood. Similarly, utilizing ligands targeting integrins (also highly expressed in HNSCCs) or other HNSCC cell surface ligands (e.g., Epidermal growth factor receptor which is frequently overexpressed in HNSCC) could increase the isolation and capture of rare CTCs. Biochemical-based schemes have been predominantly adopted for CTC isolation, with the CellSearch system being the only FDA-approved platform so far [17]. Nevertheless, selection bias exists, as CTCs undergoing epithelial-mesenchymal transition (EMT) reduce their expression of EpCAM and CKs, with EpCAM being particularly variable in HNSCC [18]. Conventional methods based on epithelial markers might not capture EMT-transformed CTCs and thus, might underestimate or fail to detect CTCs. To address this issue, negative selection targeting on blood cells for depletion rather than tumor cells for enrichment has been proposed, as CTCs do not express blood cell markers such as CD45 and CD235a [19]. Despite efforts to optimize CTC capture efficiency, satisfying outcomes in cell purity is yet to be achieved [20].

Recently, many new platforms have emerged to overcome the abovementioned challenges. Label-free inertial microfluidics approaches, for example, can avoid underestimation of CTC exhibiting downregulation of surface marker expression, and have been reported to be able to detect a larger CTC pool (3–133 CTC/mL of blood) than what had been previously achieved by the CellSearch system [21]. Researchers have also developed a 2-step CTC isolation and purification method, integrating a negative selection-based CTC isolation scheme and a subsequent 8-day spheroid cell culture for the further purification of CTCs, thereby isolating CTCs with higher purity [22].

### 2.2. Circulating Tumor Cells as Prognostic Biomarkers

With advances in technologies of CTC isolation and enumeration, there is a growing amount of research focusing on the value and potential use of CTC counts in cancer prognosis and treatment response, many of which have investigated the relationship between CTC quantitative counts and survival outcomes (Table 1). In a prospective clinical follow-up study involving 48 patients with HNSCC, Jatana et al. measured CTC numbers by the negative depletion method and observed an improved disease-free survival with no CTCs present and a worse clinical outcome with >25 CTCs/mL [23]. They reported a significant correlation between CTC presence and decreased disease-free survival (DFS) for the first time in a HNSCC population. However, in a large cohort study of 144 patients with locally advanced HNSCC, results suggested that the presence of CTCs was not predictive for DFS and overall survival (OS) in the cohort. Curiously, CTC detection trended with improved DFS and OS in patients with oropharyngeal carcinomas, while being a prognostic marker of worse DFS and OS in non-oropharyngeal HNSCC patients [24], suggesting CTCs (as currently detected) remain an imperfect prognostic liquid biomarker.

Studies have also begun to illustrate the possible application of CTCs in monitoring disease status by conducting serial measurements of CTC levels. Inhestern et al. assessed and analyzed CTC counts from blood samples of 40 patients with oral cavity SCC (OSCC) before, during, and after treatment [26]. Similarly, Wang et al. investigated CTC counts of 47 HNSCC patients throughout concurrent chemoradiotherapy [27]. Both studies concluded that changes in CTC levels were highly correlative with tumor response to treatment, with persistently high CTC levels corresponding with worse prognosis and treatment response and decreasing CTC levels correlating with improved response and outcomes.

Additionally, CTC count was reported to have a predictive value for regional metastasis in head and neck cancer. In a study that included 42 patients with locally advanced HNSCC, the detection of CTC was discovered to correlate with the nodal stage as well as regional metastasis [28]. Kulasinghe et al. demonstrated in a cohort of 60 head and neck cancer patients that the presence of CTC clusters was significantly associated with distant metastatic disease [25]. Despite these initial encouraging findings, further research is undoubtedly needed to determine the role of CTC as a biomarker for treatment response and tumor state in head and neck cancer.

### 2.3. Circulating Tumor Cells as Immunotherapeutic Biomarkers

Immunotherapy has become a promising approach for the management and treatment of numerous cancers, including HNSCC, due to the emergence of immune checkpoint inhibitors [30]. There is interest in investigation of the role of CTCs in modulating disease behavior apart from its promise as a prognostic biomarker. Programmed death 1 (PD1) checkpoint inhibitors may block the PD-1/PD-L1 immune checkpoint pathway on CTCs and activate the immune system to eliminate CTCs in circulation, thus potentially reducing the risk of metastasis and disease recurrence.

PD-L1 overexpression in CTCs was detected and used for treatment response monitoring in patients with OSCC [31]. Strati et al. demonstrated (for the first time) that assessment of CTCs overexpressing PD-L1 in liquid biopsies is feasible, with implications for monitoring patients on PD1 inhibitors—and with the potential to provide important prognostic information in a prospective cohort of locally advanced HNSCC patients. By using RT-qPCR, PD-L1 overexpression was found in 24 (25.5%) of 94 patients at baseline, 8 (23.5%) of 34 patients after nonsurgical treatment, including chemoradiation, and 12 (22.2%) of 54 patients at the end of definitive treatment [29]. An application of CTCs in HNSCC was proposed such that patients with high baseline PD-L1 expression be given a monotherapy with a PD-1/PD-L1 inhibitor, and patients with low PD-L1 expression be selected for combination therapy [32]. PD-L1 overexpression at the end of treatment was also revealed as an important independent prognostic factor which correlated with shorter progression-free survival (PFS) and overall survival (OS) compared with PD-L1 negative counterparts [29]. Similarly, a study with an HNSCC cohort treated with nivolumab found that PD-L1-positive CTCs were significantly associated with worse outcomes [25]. Other immune-regulatory molecules in CTCs, including PD-L2 and CD47, were investigated as well. Researchers have suggested that expressions of these three immune-regulatory molecules were positively correlated to one another [33].

## 3. Circulating Tumor DNA in Head and Neck Cancer

### 3.1. Characteristics and Detection Techniques

Liquid biopsy techniques have also been focused on cancer-derived non-cellular components that circulate in the bloodstream. Most prominent in these studies have been circulating tumor DNA (ctDNA). Circulating tumor DNA refers to extracellular DNA (cell-free DNA) released into the bloodstream by the sloughing cancer cells, through both pathological and physiological mechanisms, including cellular apoptosis and necrosis from rapidly proliferating cancer cells [34]. cfDNA is present in noncancer states, originating from apoptotic and necrotic cells, which are then phagocytosed by macrophages and other scavenger cells, under normal circumstances. In cancerous states, ctDNA enters circulation in increased amounts when phagocytosis is exhausted or impaired within the tumor [35]. Thus, in patients with cancer, cfDNA is comprised of a combination of noncancer cell DNA from normal cells and ctDNA from cancer cells. Generally, ctDNA represents a small fraction of total cfDNA (usually <1.0%) but may vary from less than 0.1% to over 10% depending on tumor burden, cancer stage, cellular turnover, and response to therapy [36], thus making detection and quantification challenging via traditional sequencing and analysis approaches [37].

The advent of new digital technologies has helped address this challenge, as two major methods (targeted and untargeted ctDNA approaches) are being studied and optimized [6]. Targeted approaches include PCR-based technologies such as BEAMing (beads, emulsion, amplification, and magnetic) and droplet digital PCR (ddPCR), which can optimize samples with low DNA counts or concentration, e.g., ctDNA. Additionally, certain Next-generation sequencing based technologies are being utilized to help isolate and capture ctDNA, including TAm-Seq (tagged amplicon deep sequencing), CAPP-Seq (cancer personalized profiling by deep sequencing), Safe-Seq (safe sequencing system), and AmpliSeq [38,39], each with relative strengths in sensitivity and scalability [40]. Untargeted approaches include other types of NGS-based technologies, including whole genome sequencing (WGS) and whole exome sequencing (WES), which do not require any prior knowledge of molecular alteration but are comparatively less cost-effective [41].

Studies have shown that quantification of copy number aberrations (CNAs), human papillomavirus (HPV) DNA, and somatic mutations in low level ctDNA in plasma from HNSCC patients is technically feasible via TAm-Seq, AmpliSeq, ddPCR and WGS. Combined analyses contribute to a higher sensitivity, which is paramount to utility [42,43,44,45]. Despite current limitations, investigators are having success detecting and isolating minor amounts of ctDNA from body fluids, thus opening the gate to a variety of clinical applications [46]. As technological advances continue, greater sensitivity and specificity may allow for further enhanced clinical application.

### 3.2. ctDNA as a Biomarker for Disease Staging and Detection

The concept that ctDNA concentration is increased in patients with HNSCC—and correlates with disease severity—is foundational to considering ctDNA as a diagnostic and prognostic tool (Table 2). Mazurek et al. assessed total ctDNA levels in 200 patients with HNSCC, as compared to a noncancer control group. They found that the mean level of total ctDNA was higher in the HNSCC group, especially in oropharyngeal SCCs (OPSCCs), and correlated with nodal status [47]. Similarly, Bettegowda et al. evaluated the detection of ctDNA as a prognostic tool in 359 patients with 15 various cancer types, including HNSCC. By comparing patients with metastatic cancer versus nonmetastatic cancer patients, there was a clear trend with regards to metastatic stage of disease and increased ctDNA quantity [48]. Several feasibility studies have shown that in virally-mediated cancers (e.g., in the head and neck, HPV-related oropharyngeal cancer, and Epstein-Barr virus-mediated nasopharyngeal cancer), circulating HPV and EBV DNA from either plasma or saliva is useful for diagnosing disease at an earlier stage [44,49,50]. This highlights the potential utility of tumor-specific DNA in liquid biopsy for pretreatment phases for head and neck cancer detection and staging, as well as in screening of at-risk populations.

### 3.3. ctDNA as a Biomarker for Post-Treatment Surveillance

In the posttreatment setting, ctDNA can be used to assess residual disease and stratify patients at variable risk of recurrence following curative-intent therapy. Monitoring disease by ctDNA has increasingly been investigated in a number of malignancies, including breast and colorectal cancers [53,54]. In studies by Wang et al. and Ahn et al., HPV ctDNA from posttreatment HNSCC patients were also analyzed with promising results, providing the possibility of predicting disease recurrence using ctDNA [44,49]. In another study, Hamana et al. compared preoperative and postoperative levels of ctDNA in 64 patients with OPSCC. The number of patients with tumor-specific ctDNA dropped from 40% (28/64) to 20% (13/64) after treatment; moreover, among the 28 patients with measurable ctDNA preoperatively, those with no detectable ctDNA postoperatively had no disease recurrence, whereas 4 patients with postoperative detectable ctDNA subsequently developed distant metastases [55]. Egyud et al. studied the utility of ctDNA as a diagnostic tool for recurrence in 8 HNSCC patients. Their findings showed that two of four patients with recurrence had detectable plasma ctDNA prior to clinical manifestation [51].

Longitudinal monitoring of ctDNA may allow for repeated quick, noninvasive means to assay disease status at multiple timepoints [56]. A recent prospective clinical trial with 115 HPV-positive OPSCC patients measured longitudinal HPV ctDNA levels. Kinetic data measured during curative-intent chemoradiotherapy for a median time of 23 months demonstrated that all patients with recurrence had at least two consecutive abnormal plasma HPV ctDNA levels. Furthermore, of the 87 patients with undetectable HPV ctDNA after completion of chemoradiation, none had recurrences, thus showing high sensitivity and specificity for HPV ctDNA in identifying recurrent HPV-associated oropharyngeal HNSCC [52]. ctDNA has also been investigated as a tool to augment clinical decision-making in determining radiologic presence of disease. In a study by Rutkowski et al., patients with incomplete radiographic response and elevated HPV ctDNA levels were demonstrated by PET-CT to have recurrences, suggesting that more recurrent diseases may be discovered by combining ctDNA with radiographic examination, as opposed to radiographic imaging alone [57]. ctDNA levels were also compared with metabolic response (represented by PET-CT), and their predictive values for assessing treatment response were similar [58]. Thus, the ability to assess treatment response in HNSCC may be enhanced by combining ctDNA data from liquid biopsy with standard imaging results.

## 4. Exosomes in Head and Neck Cancer

### 4.1. Characterization and Detection Techniques

Exosomes (EXOs) are defined as a subset of extracellular vesicles (EVs) within a specific size (30–150 nm), released by cells, and detectable in most bodily fluids (e.g., saliva and blood) [59,60]. EV populations are membrane-bound vesicles consisting of various types of small vesicles. These include microvesicles and EXOs, which are distinguished by their biogenesis process and biophysical properties [61]. EXOs have a lipid bilayer structure containing proteins, miRNA, mRNA, and DNA that allows for intercellular communication. The lipid bilayer also contributes to the tumor microenvironment and immune response of HNSCC [59,62]. Excitingly, EXOs have recently been introduced as potential candidates for liquid biomarkers in cancer in general, and in HNSCC in particular.

Multiple methods have been developed for EXO isolation, based on either biophysical properties (e.g., size, solubility, and density) or immune affinity [63]. Ultracentrifugation and precipitation, for example, are common protocols, based on sedimentation velocity and solubility of particles, respectively. They yield a high volume of EXOs, but are time-consuming and challenged by contamination and purity. Immunoaffinity capture methods select and isolates EXOs by recognizing specific surface antigens. They are less commonly used, owing to their relatively limited sample capacity, low yields, and high cost [46]. Clinical applications require a feasible and fast technique that can harvest EXOs with high purity and output. Therefore, researchers have established new platforms, including mini-size exclusion chromatography (mini-SEC), to further enable reproducible isolation [64].

### 4.2. EXOs as a Biomarker for Disease Staging and Detection

Exosomes have had significant recent investigations into their utility as a biomarker representing disease state in HNSCC (Table 3). Studies demonstrating EXO expression patterns and functions regulating the tumor microenvironment [65]—and EXO concentration correlating with disease activity and tumor stage—are laying down the foundation for further investigations concerning head and neck cancer [66].

Immune checkpoint antibody blockade of the PD1/PD-L1 pathway (blocking cancer cell suppression of functional T cells, allowing for T cell mediated antitumor recognition and response) is a recent advance in treatment for advanced, recurrent, and metastatic HNSCC [71]. Notably, EXOs can carry PD-L1, with implications on tumor microenvironment modulation and disease progression. Theodoraki et al. isolated EXOs from plasma of 40 patients with HNSCC and tested soluble PD-L1 by ELISA, finding that EXOs carried biologically active PD-L1. Exosome-bound PD-L1 correlated with tumor stage and lymph node status. This fundamental research suggested that circulating PD-L1 expression on EXOs can be used as a biomarker, as the levels of PD-L1 carried by EXOs were associated with disease progression in HNSCC patients [67].

MicroRNAs (miRNA) in blood and saliva have been noted to be concentrated in EXOs, with the ability to mediate cellular translational suppression, mRNA degradation and transcriptional regulation [72]. Langevin et al. isolated EXOs from four patients with HNSCC and compared the transcript levels of miRNAs in EXOs from noncancer patients and control cells. Their results showed that some EXO miRNA (including miR-486-5p, miR-486-3p, and miR-10b-5p)—secreted solely by cancer cells in culture—were substantially elevated, suggesting that specific EXO miRNAs may be promising biomarkers in HNSCC [73]. Other studies focusing on miR-21 showed that the EXO miR-21 levels correlated with tumor stage and lymph node metastasis in oral, laryngeal, and esophageal SCC patients [68,74,75]. These studies emphasized the clinical impact of EXO miR-21 as a valuable biomarker for HNSCC progression.

### 4.3. EXOs as a Biomarker for Post-Treatment Surveillance

Researchers also have been exploring the potential use of EXOs in the posttreatment surveillance phase for HNSCC. Theodoraki et al. detected plasma EXOs and tested their levels of E-Cadherin, N-Cadherin and TGF-β1 by flow cytometry. EXOs obtained pre-treatment with photodynamic therapy had a mesenchymal profile, enriched N-Cadherin and TGF-β1, and enhanced tumor proliferation, migration and invasion. EXOs collected after photodynamic therapy had an epithelial profile, carried E-cadherin, and inhibited proliferation, migration and invasion, suggesting EXO composition and their molecular cargo may serve a role in monitoring a patient’s response to therapy [69]. In another study, 18 HNSCC patients receiving a combination of cetuximab, ipilimumab, and radiation therapy were serially monitored for tumor-associated EXOs and T cell-derived EXOs. Theodoraki et al. compared the molecular cargo of EXOs between patients who developed recurrent disease and those who remained disease free. Total EXO protein and total EXO ratios increased in disease-free patients, with total CD3+, CD3(−)PD-L1+ CD3+ 15s+ (Treg-derived) EXOs elevated from the baseline levels. These levels decreased in recurrent patients, supporting the potential role of EXOs as biomarkers for posttreatment surveillance and early detection of recurrence [70].

## 5. Other Liquid Biomarkers in Head and Neck Cancer

### 5.1. RNA as a Liquid Biomarker in Head and Neck Cancer

As noted above, miRNA shows promise as a liquid biomarker in head and neck cancer. Meanwhile, many studies investigating miRNA have focused on it as cargo within EXOs (as noted above). There are additional studies looking at plasma free miRNA, mRNA and lncRNA as liquid biomarkers in cancers, including HNSCC [76]. For instance, Lu et al. [77] demonstrated (with high sensitivity and specificity) that there was significantly higher expression of plasma miR-196a and mi-196b in patients with precancerous and cancerous oral cavity lesions compared to normal controls. In addition, Liu et el. reported a significant elevation in plasma miR-31 from OSCC patients, as well as a significant reduction after tumor resection—suggesting the feasibility of applying miRNAs for diagnostic uses [78]. In a similar fashion, Mayda et al. [79] investigated GAS5 lncRNA plasma levels in 41 patients with head and neck cancer, demonstrating that persistently elevated levels of this lncRNA were associated with persistent disease, with reasonable sensitivity and specificity. Combined, these data—while preliminary—are encouraging for further investigation of RNA as liquid biomarkers in head and neck cancer, whether associated with EXOs or as free plasma biomarkers.

### 5.2. Metabolites as Liquid Biomarkers in Head and Neck Cancer

Among liquid biomarkers for cancer, tumor metabolites have had a rich history of investigation. Several metabolomic studies have been undertaken recently to identify specific metabolites or metabolomic signatures and their association with HNSCC. For instance, Shahid et al. identified that (among 3326 evaluated metabolites) stearyl alcohol and sucrose were predictive markers between OSCC patients and noncancer patients [80]. There have been encouraging reports in other squamous cell carcinomas (esophageal, cervical) suggesting the potential utility of plasma lysophosphatidylcholines as sensitive and specific cancer liquid biomarkers [81,82]. Notably, there has been recent interest in studying metabolites as cargo in EXOs, as a potential further refinement as a cancer biomarker, including in preliminary cohorts of HNSCC patients [83]. As existing technology for detection of metabolites is fairly well-established and can be relatively cost-effective, this is a field of additional opportunity for refinement and clinical validation in head and neck cancer patients.

## 6. Current Challenges and Future Directions

In recent years, there has been tremendous advancement in the field of liquid biopsy for cancer in general, and head and neck cancer in particular. Critically, the development and refinement of isolation techniques for the major liquid biopsy targets (CTCs, ctDNA, EXOs; Table 4) have paved the way for preliminary studies investigating their utility in clinical scenarios. Nevertheless, there remain significant hurdles to overcome to increase the implementation and utility of liquid biomarkers in clinical care in HNSCC.

Currently, all major liquid biopsy techniques face challenges; they have not yet achieved optimal sensitivity and specificity at a level acceptable for clinical use. Further refinement of platforms allowing for enhanced tumor-associated liquid biomarker capture will be critical to optimizing both sensitivity and specificity of assays, including immune affinity and label-dependent capture of CTCs and EXOs or ddPCR for ctDNA.

A critical challenge for all liquid biopsies is the ability to truly target and separate the cancer-associated biomarker from existing circulating cells, DNA and EXOs that are generated from noncancer tissues. As noted by the studies above, there is an opportunity for high specificity if the biomarker target is not endogenous or expressed by normal tissue, e.g., with HPV and EBV viral markers in appropriate virally-associated head and neck cancers. For nonvirally-associated HNSCCs, there are no ubiquitous mutations, surface markers, or other profiles that are completely specific to HNSCC. While studies, as noted above, have highlighted potential commonly altered profiles (e.g., mi-R21 for EXOs, TP53 or PIK3CA genetic mutations in CTCs, or ctDNA), these are far from universal. Future identification and validation of multiplex expression profiles (e.g., gene probes targeting ctDNA harboring any of a number of common mutations in HNSCC, targeting a combination of miRNA or tumor-associated surface markers on EXOs) may allow for increased sensitivity and specificity for isolating true cancer-associated liquid biomarkers.

While blood-based liquid biopsies have been the most common avenue of investigation, HNSCCs offer a unique opportunity for further refinement of salivary or oral rinse-based liquid biopsies, given that these cancers are of upper aerodigestive mucosal origin and can shed tumor cells, tumor DNA, and EXOs directly into oral and upper airway secretions. Indeed, further studies comparing the efficacy of salivary versus blood-based liquid biopsies (or combinations thereof) will be of great interest. Additionally, researchers are continuing to look for new liquid-based biomarkers other than the CTCs, ctDNA, and EXOs discussed above; for instance, in addition to RNA and metabolite biomarkers, tumor-educated platelets represent another promising liquid biopsy candidate [84].

As liquid biopsies in HNSCC increase in their sensitivity, specificity, and overall reliability, consideration of their incorporation into clinical trials will be of critical importance. As noted above, integrating these tests in order to augment current evaluations of disease status (as with radiologic response rates) may be a feasible initial design. With increased reliability, further treatment stratification based on liquid biomarker findings may be future solutions. Treatment algorithms can be in neoadjuvant settings (where currently archaic radiologic and clinical exams are our primary tumor response assessment methods), or in the adjuvant or surveillance setting (where it is currently challenging to detect any microscopic residual disease). Liquid biopsy-dependent trial design may be most achievable in EBV and HPV-related head and neck cancers, where the EBV and HPV DNA tests have been more vigorously investigated and validated. Indeed, clinical trials for nasopharyngeal cancer are already being launched that utilize EBV DNA for treatment stratification. The use of different high-throughput analytical platforms—as well as different biomarkers and capture systems—also highlights the need for interventional studies and large-scale clinical trials for validation and standardization [85,86,87].

## 7. Conclusions

Liquid biopsies—as a biomarker for disease detection, prognostication, and therapeutic intervention in head and neck cancer and cancer in general—are an alluring field of research. They hold great potential as a noninvasive, rapid, repeatable adjuvant tool for cancer detection and surveillance. As we rapidly approach an era with increased treatment stratification and personalization of cancer care, liquid biopsies may play a critical role in facilitating personalized management of head and neck cancer patients. Although there are current challenges—including limited specificity, sensitivity and lack of standardization—their potential applications are rapidly expanding. In this review, we summarized the three most studied liquid biopsy biomarkers (CTCs, ctDNA and EXOs) for HNSCC. We examined the current state of their research, as well as challenges and opportunities for improvement for each technique. Undoubtedly, further studies and trials are imperative for liquid biopsies to be incorporated into standard of care clinical practice. Nevertheless, with dramatic recent advances in this field, the study of liquid biopsies in head and neck cancer will be of continuing great value and interest.

## Figures and Tables

**Table 1:** Key studies investigating circulating tumor cells (CTCs) as biomarkers in head and neck squamous cell carcinomas (HNSCCs).

Population/Trial Design	Detection Methodology	Primary Outcome	Major Findings	Study Strengths	Study Limitations	References
HNSCC undergoing definitive surgery	Negative depletion followed by positive staining (CK)	Disease-free survival (DFS)l	Patients with no detectable CTCs had significantly higher DFS	Patients treated homogenously with surgery	Single timepoint study, drawn during surgery	[23]
Stage III-IV HNSCC after definitive surgery, undergoing adjuvant (chemo)radiation (CRT)	Positive staining (EGFR)	1. DFS 2. Overall survival (OS)	1. CTCs detectable in 29% of patients 2. Non-OPSCC HNSCC patients with detectable CTCs have worse DFS and OS	1. Patients treated homogeneously with surgery, followed by adjuvant therapy 2. Large cohort (*n* = 144)	1. Relying on EGFR positivity to define CTC 2. Single timepoint study	[24]
Treatment-naïve HNSCC	ClearCell FX system Negative depletion (CD45) Positive staining (CK)	Progression-free survival (PFS)	1. CTCs detectable in 47.8% of patients 2. Patients with detectable CTCs have worse PFS 3. PD-L1 positive CTCs associated with worse survival	Analysis of multiple expression markers (PD-L1, ALK, EGFR)	1. Small cohort (*n* = 23) 2. Single timepoint study 3. Mixed study with various stages, treatments	[25]
Advanced OSCC after induction chemotherapy, prior to definitive treatment	Positive staining (EpCAM)	1. Recurrence-free survival (RFS) 2.OS	1. CTCs detectable in 80% of patients 2. Higher baseline CTC and maximal CTC associated with worse RFS and OS	1. Phase II trial with defined induction chemotherapy protocol 2.Serial timepoints 3. Homogeneous population of OSCC undergoing treatment	1. Protocol not reflecting general standard of care 2. Smaller cohort (*n* = 40)	[26]
HNSCC patients undergoing definitive CRT	Negative depletion	1. PFS 2. OS	1. CTC reduction associated with response to chemoradiation and improved PFS and OS	Serial timepoints	Cohort limited to chemoradiation patients	[27]
Advanced HNSCC undergoing definitive CRT	Negative depletion (CD45) Positive staining (CK, EpCAM, EGFR)	Detecting metastases	1. CTCs detected in 42% of patients 2. N2b or higher stage associated with higher CTCs	1. Robust protocol for CTC delineation 2. Serial timepoints	1. Small cohort (*n* = 26) 2. Cohort limited to chemoradiation patients 3. No survival analyses	[28]
Advanced HNSCC undergoing induction chemotherapy, followed by definitive CRT	Positive staining (EpCAM)	1. PFS 2. OS	PD-L1 overexpression on CTC associated with worse PFS and OS	1. Large cohort (*n* = 113) 2. Investigation of PD-L1 as biomarker	Cohort limited to chemoradiation patients	[29]

**Table 2:** Key studies investigating circulating tumor DNA (ctDNA) as biomarkers in HNSCC.

Population/Trial Design	Detection Methodology	Primary Outcome	Major Findings	Study Strengths	Study Limitations	References
Asymptomatic population screening	RT-PCR for plasma EBV ctDNA	1. Rate of EBV DNA positivity 2. Rate of NPC in positive EBV DNA patients 3. Sensitivity, specificity of EBV DNA for NPC	1. 5.5% detectable EBV DNA rate 2. 11% confirmed NPC among EBV DNA positive patients 3. 97.1% sensitivity, 98.6% specificity for NPC	1. Robust population study (*n* > 20,000) of endemic population for NPC 2. Demonstration of utility of EBV DNA as a screening test	1. False positive rates for EBV DNA 2. Challenges to apply to non-endemic populations	[50]
HNSCC before definitive or salvage therapy	PCR (HPV, *TP53, PIK3CKA, CDKN2A, FBXW7, HRAS, NRAS* mutations) for plasma and/or saliva ctDNA	1. Rate of ctDNA detection in saliva 2. Rate of ctDNA detection in plasma	1. 96% ctDNA detection rate when both plasma and saliva tested 2. 100% saliva ctDNA identified in OSCC patients	1. Demonstration of ability to identify salivary ctDNA 2. High detection rates demonstrated 3. Demonstration of detection of non-virally mediated HNSCC	1. Hetergeneous population of HNSCC patients 2. Not all patients obtained saliva and blood testing 3. No survival analyses	[44]
OPSCC before and after definitive therapy	RT-PCR for plasma and saliva HPV ctDNA	1. Negative predictive value (NPV), positive predictive value (PPV), sensitivity, specificity of HPV ctDNA for tumor detection	1. Sensitivity 76% 2. Specificity 100% 3. NPV 42% 4. PPV 100%	1. Serial timepoints 2. Demonstration of post-treatment ctDNA positivity correlation with worse survival	1. Protocol limited to HPV-related HNSCC 2. Poor NPV	[49]
HNSCC before and after definitive therapy	Next-generation sequencing of plasma ctDNA to compare to identified primary tumor DNA mutations	Baseline ctDNA mutation rates correlating with primary tumor	75% plasma ctDNA mutation rate	Demonstration of utility of mutational profiling with primary tumor	1. Small cohort (*n* = 8) 2. No evidence of correlation with ctDNA positivity and recurrence	[51]
p16 positive OPSCC after definitive CRT	ddPCR for HPV ctDNA	NPV, PPV of HPV ctDNA for cancer surveillance	1. NPV 100% 2. PPV 94%	1. Large cohort (*n* = 115) 2. Serial timepoints 3. Demonstration of utility of HPV DNA as a surveillance test	1. Protocol limited to HPV-related HNSCC	[52]

**Table 3:** Key studies investigating exosomes (EXOs) as biomarkers in HNSCC.

Population/Trial Design	Detection Methodology	Primary Outcome	Major Findings	Study Strengths	Study Limitations	References
HNSCC	Size exclusion chromatography, CD63 staining	1. EXO PD-L1 levels 2. Correlation of EXO PD-L1 levels with clinicopathologic disease	High EXO PD-L1 correlated with advanced and active disease	Validation of ability to test EXO for marker expression	1. Heterogeneous HNSCC population 2. Small subpopulations, correlations preliminary	[67]
Laryngeal SCC (LSCC)	CD63 staining	1. Correlation of EXO miR-21 and HOTAIR levels with LSCC	High EXO miR-21 and HOTAIR expression correlated with LSCC	Validation of EXO to differentiate benign and malignant processes	1. Limited to LSCC cohort 2. No prognostic or survival analyses	[68]
HNSCC before and after photodynamic therapy	Size exclusion chromatography, CD63 staining	EXO E-Cadherin, N-Cadherin and TGF-β1 levels	Pre-treatment EXO levels enriched in N-Cadherin and TGF-β1	Investigation of EXO cargo as markers for epithelial-mesenchymal transition	1. Small cohort (*n* = 9) 2. Non-standard of care treatment 3. Preliminary data	[69]
HNSCC undergoing CRT with ipilimumab	Size exclusion chromatography, CD63 staining	1. DFS 2. EXO changes during therapy	Total EXO levels, CD3 + CTLA4_ EXOs decreased after ipilimumab	1. Phase I clinical trial setting 2. Serial timepoints	1. Small cohort (*n* = 18) 2. Clinical trial treatment population	[70]

**Table 4:** Current strengths and limitations of major liquid biopsy targets.

	CTC	ctDNA	EXO
**Detection Principles**	1. Label-dependent methods 2. Label-independent methods	1. Targeted approaches 2. Untargeted approaches	1. Immune affinity based methods 2. Biophysical property-based approaches
**Biofluid Concentration**	Very Low	Variable/Moderate	High
**Detection in Biofluids**	Blood	Blood, saliva	Blood, saliva, urine, sweat
**Sensitivity**	Low	Variable/Higher	Higher
**Specificity**	Variable	Variable/Higher	Variable/Low
**Clinical Readiness/Trials in Head and Neck Cancer**	1. Early phase data 2. FDA-approved test in other cancers	Completed and ongoing trials with EBV, HPV DNA	Not yet
**Other Characteristics**	+Can perform studies on morphology and gene profile +Immune checkpoint markers on CTCs allowing immunotherapeutic studies	+Short half-life +Able to detect genetic mutations	+Short half-life +Can analyze variety of molecular cargo (e.g., miRNA, metabolites) −Lack of standardization for detection and isolation

## Data Availability

Not applicable.
